# Viral Vectors for the Induction of Broadly Neutralizing Antibodies against HIV

**DOI:** 10.3390/vaccines7030119

**Published:** 2019-09-19

**Authors:** Sarah Wilmschen, Joern E. Schmitz, Janine Kimpel

**Affiliations:** 1 Division of Virology, Medical University of Innsbruck, Innsbruck 6020, Austria; 2 Center for Virology and Vaccine Research, Beth Israel Deaconess Medical Center, Harvard Medical School, Boston, MA 02115, USA

**Keywords:** HIV vaccine, neutralizing antibodies, viral vector vaccine, Env

## Abstract

Extensive research on generating an efficient HIV vaccine is ongoing. A major aim of HIV vaccines is the induction of long-lasting, broadly neutralizing antibodies (bnAbs) that can confer sterile immunity for a prolonged period of time. Several strategies have been explored to reach this goal, i.e. protein immunization, DNA, or viral vectors, or a combination thereof. In this review, we give an overview of approaches using viral vectors for the induction of HIV-specific bnAbs. Many pre-clinical studies were performed using various replication-competent and -incompetent vectors. Amongst them, poxviral and adenoviral vectors were the most prevalent ones. In many studies, viral vectors were combined with a DNA prime or a protein boost. However, neutralizing antibodies were mainly induced against the homologous HIV-1 vaccine strain or tier 1 viruses, and in rare cases, against tier 2 viruses, indicating the need for improved antigens and vaccination strategies. Furthermore, we also review next generation Env antigens that are currently being used in protein vaccination approaches and point out how they could be utilized in viral vectors.

## 1. Introduction

Many vaccines are based on live-attenuated pathogens [1,2,3,4,5,6]. These vaccines usually induce very robust and long-lasting immune responses and are also easy to produce at relatively low costs [7]. However, for viruses such as HIV, live-attenuated vaccines do not have a safety profile that is acceptable for human use and bear the risk of reverting to a non-attenuated, pathogenic phenotype due to the high mutation rate of host cellular cytidine deaminase and the viral reverse transcriptase [8]. Here, heterologous viral vector vaccines are a promising alternative with a favorable safety profile. Viral vaccine vectors can be either replication-defective or replication-competent. Especially for replication-competent vectors, many of the advantages of live-attenuated vaccines should also apply, such as robust and lasting immune responses, delivery of HIV Env in a favorable configuration, strong expression of the vaccine antigen in vivo, co-stimulatory immune activation due to virus associated danger signals, and an easy and cost-effective production process. One downside of viral vaccine vectors is that pre-existing or vaccination-induced anti-vector immunity might limit the efficacy of the vaccine upon boosting with the same vaccine vector. Additionally, the vector-specific immunity could also interfere with the safety of the vaccine vector. Previous studies have shown that this can become especially relevant for an HIV vaccine. In the STEP study, a large HIV vaccine efficacy trial using an adenoviral (Ad) 5 vector, an increased rate of HIV infections was observed in patient subgroups with a high pre-existing immunity against the Ad5 vaccine vector [9]. A possible explanation for this phenomenon is that pre-existing Ad5-specific CD4^+^ (cluster of differentiation 4) T cells were activated by the immunization, making them more susceptible to HIV infection [10]. In the following review, we will first discuss new Env immunogens that should have the potential to induce HIV-specific broadly neutralizing antibodies (bnAbs). Subsequently, we will review viral vectors that are currently being used and will discuss the advantages of viral vectors for the induction of bnAbs.

## 2. Design of Env Immunogens for BnAb Induction

In general, vaccine-induced immunity is provided by the induction of strong, long-lasting humoral immune responses. HIV Env, the major target for the induction of protective antibodies, is encoded as a gp160 (glycoprotein) precursor protein. Gp160 is cleaved by furin into the transmembrane subunit gp41 and the surface subunit gp120, which together build a non-covalently linked heterodimer. Three of these heterodimers form the functional Env trimer. Induction of bnAbs against HIV faces a number of problems, such as the high diversity of HIV strains; glycosylation patterns of Env, which protect vulnerable epitopes; unusual characteristics of bnAbs; lack of suitable animal models; and correlates of protection. A major problem in the generation of bnAbs following HIV vaccination is the paucity of naïve B cells that recognize the broadly neutralizing epitopes of HIV. The affinity of the B cell receptor of naïve B cells is either exceptionally low or often not existing [11]. To allow for the ranking of induced nAb responses in terms of breadth and potency, a classification into four subgroups (tier 1A, 1B, 2, and 3) is generally performed. The groups are defined as those with very high (tier 1A), above-average (tier 1B), moderate (tier 2), or low (tier 3) sensitivity to antibody-mediated neutralization [12]. Although a large number of clinical trials tested different antigens and delivery systems, none of the HIV vaccine trials conducted so far succeeded in the induction of bnAbs against primary isolates [9,13,14,15,16,17,18]. Also, in animal models, the induction of bnAbs remains challenging. However, recently, a couple of studies in animal models have resulted in the induction of autologous tier 2 neutralizing antibodies [19,20,21,22]. These include studies using native-like trimers, immunogens focusing the immune response to certain vulnerable epitopes, sequential series of immunogenes to guide antibody development in the direction of bnAbs, and immunogens that have been designed to engage germline versions of bnAbs. Currently, these approaches are mainly utilized in protein vaccination strategies. However, some of these strategies could also be applied to viral vector vaccines.

The prototype for a native-like trimer is the BG505 SOSIP.664 trimer [23]. SOSIPs are cleaved gp140 molecules held together by an additional disulfide bridge between both subunits. BG505 SOSIP.664 trimers mimic the native Env configuration and bind many known bnAbs. In animal models, SOSIP trimers induce better antibody responses than uncleaved gp140 [24]. Currently, a clinical trial is recruiting patients to test BG505 SOSIP.664 trimers in humans (ClinicalTrials.gov Identifier: NCT03699241). However, the disadvantage of expressing SOSIP variants from a viral vector is that the SOSIP configuration needs sufficient furin expression in the target cell to allow for complete cleavage, and consequently, efficient folding. As it is likely that in vivo target cells of the viral vector might not express enough furin to ensure complete cleavage, the folding of SOSIPs delivered by a viral vector might be compromised. An alternative for native-like trimers encoded by a viral vector is the native flexible linker (NFL) configuration. Here, a 10 amino acid flexible glycine-serine (GS) linker replaces the furin cleavage site [25]. It has been shown for soluble gp140 proteins that the NFL configuration, similar to the SOSIP configuration, allows for a native-like folding [25]. In a nonhuman primate model, both variants, SOSIP and NFL, induced similar titers of HIV neutralizing antibodies [26]. 

As the high diversity of HIV is one of the problems in inducing bnAbs, consensus antigens have been generated. In consensus antigens, strain specific characteristics are removed to enable the induction of antibodies with a higher neutralization breath. Example immunogens for this strategy are ConM SOSIP.v7 and ConSOSL.UFO.664, which are both consensus M trimers, the former with a classical SOSIP, the later with a cleavage-independent uncleaved pre-fusion optimized (UFO) design [19,27]. For the expression from a viral vector, the UFO design might again be favorable compared to SOSIP variants, similar to the NFL configuration, as folding is independent of the furin expression of in vivo target cells of the vector. Both consensus strategies, ConM and ConS, induced autologous tier 2 neutralizing antibodies in animal models when delivered as a protein or DNA vaccination, and clinical trials have been initiated to test them as protein immunization in patients (ClinicalTrials.gov Identifiers: NCT03816137 + NCT03961438). In one of these studies, chemically cross-linked versions of ConM and ConS are used in additional to the non-modified proteins, a strategy that obviously cannot easily be transferred to vector-encoded antigens.

Multimerization of Env is believed to enhance immune responses. For example, immunogenicity of ConM trimers was significantly enhanced when coupled to ferritin nanoparticles compared to soluble protein [27]. Multimerization can be achieved by coupling Env to nanoparticles or liposomes or by incorporating them into virus-like particles (VLPs). Also, Env that is encoded by a viral vector and incorporated into viral particles can act in the same way. In a recent study, we have shown that a viral vector expressing a particle-incorporated variant of Env induces higher antibody titers compared to the same vector expressing a secreted Env variant [28]. 

To overcome the induction of mainly strain-specific nAbs, mixes of different immunogens, either administered together or sequentially, have been used. However, this bears the risk of only inducing autologous antibodies against the immunodominant variant [29]. An alternative to this approach is sequential immunization with Env isolates from a patient at different time points after infection. The idea is to mimic natural infection by vaccination, and thereby trigger the development of bnAbs [30,31]. This strategy is currently being tested in a clinical trial with gp120 variants derived from the CH505 isolate directly after transmission (transmitted founder virus) or variants isolated from the same patient at weeks 53, 78, and 100 (ClinicalTrials.gov Identifier: NCT03220724). 

A second example for guiding antibody responses toward bnAb development is to use antigens that can engage germline versions of bnAbs. Responses are subsequently boosted using more mature immunogens. B cells, encoding HIV neutralizing antibodies, usually need to undergo an exceptionally high number of somatic hypermutations in order to produce bnAbs. Non-modified variants of native-like trimers usually do not bind well to germline precursors of bnAbs, and consequently do not trigger the expansion of corresponding B cells and the high somatic hypermutation rates that are needed [11]. To overcome this problem, immunogens with an enhanced binding to these germline B cells have been designed and these immunogens activated germline B cells in human antibody knockin mice [32,33,34,35]. Here, especially immunogens targeting germline versions of bnAbs specific for the CD4 binding site have been explored. Prototypes in this category are eOT-GT8 and BG505 GT1.1. However, studies in humans will need to show whether this concept is also valid in humans where the number of germline bnAb encoding B cells is low and the cells will have to compete with B cells encoding non-neutralizing antibodies. For eOT-GT8, a clinical trial has been initiated to analyze the safety and immunogenicity of this approach (ClinicalTrials.gov Identifier: NCT03547245). In this trial, the protein is coated on nanoparticles. However, viral vectors that encode and present germline targeting antigens on their surface are also an interesting approach that should be explored.

Although the above-mentioned studies are promising, one approach alone might not be sufficient for an effective HIV vaccine strategy. For an efficient protection, it will likely be necessary to induce more than just one bnAb against HIV. Therefore, a combination of different strategies targeting multiple epitopes simultaneously will be necessary. Xu and colleagues, for example, have combined a fusion peptide-coupled carrier protein prime with a native-like trimer boost, and thereby have enhanced antibody responses compared to the native-like trimer only [36]. When viral vectors are used for multiple immunizations, it is important that they induce, if at all, only a minor vector-specific immunity, which could otherwise limit the efficacy of sequential immunizations. As most viral vectors induce vector-neutralizing antibodies after few immunizations [37,38] or vector-specific pre-existing immunity exists [39,40], it will be necessary to combine different vectors or viral and non-viral delivery methods. Thus, the generation of an effective HIV vaccine will require the combination of different antigen and delivery strategies for the induction of bnAbs, e.g. prime with a viral vector encoding a germline targeting immunogen and boost with either the mature protein or the mature protein encoded by a different vector. 

## 3. Viral Vectors for HIV Vaccination

A huge number of different viral vectors have been explored as potential HIV vaccine candidates. These HIV vaccine candidates generally either aim for the induction of bnAbs to provide sterile immunity or for the induction of cytotoxic T cell responses to control or eliminate an infection after it has been established. The former strategy mainly uses Env as an immunogen, while T cell responses are generally induced by other HIV antigens such as Gag, Pol, and Nef. For the induction of HIV-specific T cell responses, Ad or CMV (cytomegalovirus) vectors are promising candidates as reviewed elsewhere [41]. Table 1 gives an overview of vectors used in pre-clinical studies with the aim to induce neutralizing antibodies against HIV. Table 1 contains studies matching the keywords “HIV viral vector” in PubMed. In this review, we included only studies using viral vectors for active immunization. We did not include studies that deliver HIV-neutralizing antibodies via passive immunization or vectored immunoprophylaxis (VIP). Both strategies are reviewed in detail elsewhere [42,43] and may help circumvent current problems in inducing HIV bnAbs. Passive infusion of VRC01 antibody is currently being evaluated in the Antibody Mediated Prevention (AMP) studies (HVTN 704/HPTN 085; ClinicalTrials.gov Identifiers: NCT02716675 + HVTN 703/HPTN 081; ClinicalTrials.gov Identifier: NCT02568215), which are two large phase II efficacy studies with bimonthly antibody injections over a time period of 18 months. However, the antibody half-life and the need for repeated infusions might limit practicability in rural areas. VIP means delivery of a neutralizing antibody via a viral vector. Delivery of a modified VRC07 HIV bnAb via an AAV (Adeno-associated virus) vector rendered humanized mice resistant to intravaginal challenge with a transmitted/founder virus (T/F) strain [44]. In a phase I clinical trial with an AAV, encoding the bnAb PG9, HIV neutralization was detected in the serum of four volunteers [45]. AAV-mediated VIP is not only applied for HIV, but also for other pathogens, like influenza [46]. However, VIP action in humans can be limited by an antibody response against the encoded bnAb. Another drawback for both, passive immunization and VIP, is a narrow and pre-defined repertoire of neutralizing antibodies. Therefore, it might be necessary to use a cocktail of antibodies/vectors to prevent escape mutations. In contrast, active immunization with viral antigens provides a more flexible approach and might generate a broader response.

When using viral vectors for vaccination, potential advantages and disadvantages of the vector have to be weighed against each other. The ideal viral vaccine vector has a good safety profile; is not connected to any disease in humans; shows no or low pre-existing immunity in the target population; can easily be produced at low costs under GMP (good manufacturing practice) conditions; can easily be modified to accommodate foreign vaccine antigens; is genetically stable; and can easily be stored, transported, and administered.

Although many different vectors have been used in pre-clinical models, only canarypox and Ad vectors have so far been tested in large clinical efficacy studies [9,47,48]. While the RV144 trial, combining a canarypox vector with a gp120 protein boost, showed a moderate efficacy of 31.2% after three years, all studies using Ad vectors (STEP, Phambili, HVTN505) failed to provide protection [9,49]. Alternative Ad vectors are currently being evaluated in a Phase IIb efficacy study (HVTN 705/HPX2008, ClinicalTrials.gov Identifier: NCT03060629) in order to determine whether an Ad26-based HIV vaccine approach is more suitable for the induction of T cell responses and will hopefully lack the side effects observed with Ad5 vectors. The RV144 trial aimed to induce bnAbs using Env as immunogen. However, bnAbs were not induced in the vaccinees but non-neutralizing binding antibodies were determined to be the major correlate of protection [50]. Whether a follow-up study (HVTN 702, ClinicalTrials.gov Identifier: NCT02968849) will lead to more effective and durable immune responses than the RV144 trial remains to be seen when the currently ongoing study is going to be unblinded and evaluated.

## 4. Viral Vectors for the Delivery of Next-Generation Env Antigens

Viral vectors are a promising platform for the delivery of next-generation Envs, such as native-like trimers and germline-binding Env variants. Some special characteristics of viral vectors might help to overcome the limitations of past HIV vaccine strategies. 

Attenuation and replication competency are important aspects with regard to viral vectors. On the one hand, replication competence is favorable, as it induces longer lasting transgene expression, as it was shown for Ad vectors [96]. Additionally, the viral vector-mediated adjuvant effect is potentiated due to the constant triggering of innate immune receptors. On the other hand, replication competence might be associated with safety concerns, such as a release of genetically modified organisms into the environment, integration into the genome, and uncontrolled replication. To avoid the risk of uncontrollable replication in a human vaccine, viruses obtained from nonhuman natural hosts are explored as vaccine vectors, such as fowlpox that replicates only in avian cells [69] or simian Ad [78]. Another approach is the use of a semi-replication system, which brings the advantages of replication, increased transgene expression, and safety [97]. This approach, however, has not yet been applied for the induction of HIV-specific nAbs.

Using replication-competent vectors is only possible for some viruses and many vectors for vaccine development had to be modified to abrogate replication competency and consequently improve the safety profile. Many poxviral vaccine strains, for example, were generated by repeated in vitro passaging of the virus. In the case of the modified vaccinia virus Ankara (MVA), the virus was passaged more than 570 times on chick embryo fibroblasts, leading to large deletions in the viral genome and avirulence in a variety of mammalian cells [98]. The modified Copenhagen strain NYVAC (New York vaccinia virus) was generated by deleting 18 additional open reading frames from the Copenhagen vaccine strain, leading to a highly attenuated virus [99]. Both are widely used for HIV vaccine approaches (see Table 1). However, it has recently been shown that an NYVAC variant, NYVAC-C-KC, where replication competency had been restored by reincorporating the *K1L* and *C7L* host range genes, induced enhanced HIV-specific immune responses in rhesus macaques [59]. Ad vectors, one of the most intensively studied viral vectors for gene delivery and vaccination, commonly have a deletion of the E1 gene and often additionally of other early genes that render them replication-incompetent. However, some studies use replication-competent simian Ad vectors and there is evidence that these replication-competent vectors induce improved immune responses [78]. Some vectors, such as the vesicular stomatitis virus (VSV), need to be attenuated to remove their inherent neurotropism [100]. One strategy to abrogate the neurotropism but keep replication competency was to relocate the glycoprotein G of VSV to the end of the viral genome [51]. Alternatively, VSV-G was replaced by the transgene of choice (e.g. the Zaire Ebola virus glycoprotein in the case of the VSV-ZEBOV Ebola virus vaccine [101]) or with a non-neurotropic glycoprotein from another virus [28,102]. 

Induction of HIV bnAbs is difficult and requires an exceptionally high number of somatic hypermutations [103]. Somatic hypermutation is antigen-dependent, will only occur in activated B cells, and requires T cell help. A prolonged delivery of the vaccine antigen either via an osmotic pump, releasing the protein and adjuvant over a two-week interval, or via a two-week escalating dose injection scheme enhanced the immune responses in mice and nonhuman primates [104,105,106]. These improved immune responses might be explained by several mechanisms, such as an enhanced availability of structurally intact antigen, a prolonged T cell help, and an improved immune complex formation on follicular dendritic cells [107]. While osmotic pumps and frequent injections of vaccine antigens are not practicable for large-scale use as an HIV vaccine, viral vectors could be a promising alternative in this regard. Viral vectors, especially replication-competent vectors, will produce high amounts of vaccine antigen in vivo over several days and might thereby simulate this situation. Additionally, the general immune activation caused by many viral vectors might help to stimulate somatic hypermutation and consequently improve antibody quality. 

A strong advantage of viral vectors compared to protein vaccination and non-viral delivery systems in general is that viral vectors are “self-adjuvanted” [108]. Viral replication activates innate immune sensors, such as pattern recognition receptors, and thereby creates a pro-inflammatory environment via signaling through NFκB (nuclear factor kappa-light-chain-enhancer of activated B cells) and MAP (mitogen-activated protein) kinase pathways. Another important innate defense mechanism of cells is the type I interferon (IFN) system. Upon infection, type I IFNs are induced, which transfer the infected and neighboring cells into an antiviral state, for example, by induction of MxA (Myxovirus resistance protein A), ISG-15 (interferon-stimulated gene 15), PKR (protein kinase R), and the 2-5A synthetase/RNase L systems. All of this can help to enhance the induction of adaptive immune responses, and, thereby, might increase the durability of responses upon vaccination with viral vectors. Depending on the level of attenuation/replication competency of the vector, it might still be necessary to use adjuvants (not covered in this review). However, some attenuated or replication-incompetent vectors have a very strong adjuvant effect due to the deletion of viral immunomodulators, and thereby induce stronger vaccine antigen-specific immune responses than their non-attenuated counterparts. For example, most recombinant Ad vectors have a deletion of the E3 gene, which is involved in evading host immunity [109]. Also, poxviral vectors, such as NYVAC and MVA, have deletions in immunomodulatory genes. The deletion of *B19R*, an inhibitor of the type I IFN response, in NYVAC was found to augment immune responses [57]. 

Deletion of viral genes not only affects the induced immune responses and the safety profile of the vector, but for some vectors, it is also necessary to allow for the expression of several foreign vaccine antigens by the viral vector. Viruses with a small genome only tolerate limited additional transgenes, for example, ≈4 kb for VSV. For some vectors, such as AAV, it is necessary to delete large parts of the viral genes. Even then, AAV can only accommodate ≈4.5 kb of foreign sequences. Deletion of the E1 and E3 genes allows for packing of 7–8 kb of transgenes in Ad vectors, and additional deletion of the E4 gene further increases packing capacity. Poxviral vectors, like MVA or NYVAC, have with 25–30 kb by far the largest capacity for foreign transgenes [110]. For some viruses, such as VSV, these packaging capacities can be overruled, leading to an elongated virus capsid. However, this also leads to attenuation of the vector. Increasing the genome size might not only influence replication fitness, but can also affect genetic stability, as shown for Ad [111,112]. In vitro passaged MVA and NYVAC were found to be very stable [113], while transgene stability in VSV vectors was found to be dependent on its position in the genome [114].

For protein vaccination, native-like trimers are usually used as soluble gp140 molecules. However, this comes with the potential disadvantage that non-neutralizing, immunodominant antibodies against the base of the trimer are potentially preferentially induced [104,115]. Non-neutralizing antibodies have been shown to be capable of impairing HIV infection and replication. However, this is believed to occur with a much lower efficacy than for bnAbs [116]. Additionally, many known bnAbs, such as 2F5 or 10E8, require binding to the membrane for optimal binding to HIV Env [117,118], indicating that membrane-anchored Env should have advantages compared to soluble Env. Viral vectors are good candidates for delivering membrane-anchored Env. This Env can be expressed on the surface of infected cells and ideally additionally incorporated into the vaccine virus particles. Presentation of membrane-anchored Env in a favorable conformation should improve antibody titers and the quality of antibodies. There are several examples that have shown that a membrane-anchored Env induces higher antibody titers than the soluble form [28,72]. Only a few viral vectors, such as VSV and Newcastle disease virus, allow for the incorporation of Env into the viral membrane [28,51,119]. Such particle-incorporated Env induced higher antibody titers compared to Env that was only presented on the cell surface or to secreted Env [28]. This is not only true for enveloped viruses, where Env is incorporated into the viral membrane, but also for non-enveloped viruses, such as Ad vectors, where Env is fused to the capsid protein, and thereby becomes a part of viral particles. Presentation of Env on the viral particles might have several advantages for the induction of Env-specific antibodies such as the multimerization of Env and a longer half-life. Env incorporated in the membrane of vaccine vectors might fold into a native-like conformation as on HIV particles. Additionally, T cell help for HIV-specific antibodies might be enhanced for particle-incorporated Env due to intrastructural help [120,121].

## 5. Conclusions

The HIV field has made great progress in determining potential correlates of protection in the humoral, cellular, and innate immune system. However, once HIV infection is established in humans, the immune system is apparently not capable in eradicating the infection, similar to observations made in nonhuman primate natural hosts of simian immunodeficiency virus (SIV) infection. Thus, prevention of HIV infection is a major focus in the worldwide efforts to combat this disease. In recent years, the discovery of neutralizing epitopes on HIV and neutralizing/broadly neutralizing antibodies against HIV has advanced the field significantly. While an effective HIV vaccine is still elusive, the field is slowly moving forward to generate humoral immune responses via HIV vaccination strategies that hopefully become broad enough to cover the enormous diversity of HIV strains.

Live viral vectors are promising candidates for the delivery of next-generation Env antigens and future preclinical and clinical studies will show whether they can achieve the induction of bnAbs and how durable the induced antibody responses are. However, limitations, such as anti-vector immunity and the need for different bnAb specificities, will make it necessary to utilize viral and non-viral delivery systems in heterologous prime/boost regiments. Furthermore, it might be necessary to combine antibody strategies with the efficient induction of T cell responses to achieve long-lasting protection against HIV.

## Figures and Tables

**Table 1:** Viral vectors in pre-clinical studies.

Virus Family	Strain ^i^	Type Env & Strain/Clade	Specificity nAbs Induced ^ii^	Dose ^iii^; Organism; Route ^iv^	Combination	Ref.
Rhabdovirus	VSV	gp140: G ^v^, clade B	SF162.LS (clade B)	10^7^ pfu; mice; i.n. & i.m.	-	[51]
		gp120: G ^vi^, HXB2	Homologous	10^6^ pfu; mice; i.n.	-	[52]
		gp140: G, 89.6	Homologous	10^5^–10^6^ pfu; mice & macaques; i.n. i.m. i.p.	-	[53,54]
	VSV + rabies	gp140: G ^vii^	Laboratory-adapted strain (HIV-1_MN_)	(3–4) × 10^5^ ffu (RV) or pfu (VSV); i.m.	-	[55]
	VSV-GP	gp140: G, 1086.C	Tier 1A clade C	10^7^ TCID_50_; mice, rabbits; i.m.	-	[28]
	Rabies	gp160, 89.6 & NL4-3	Homologous	10^6^ ffu; mice; s.c.	Protein	[56]
Poxvirus	NYVAC-C-KC (ΔB19R ^viii^)	gp140, ZM96	Tier 1; tier 2 clade C	10^8^ pfu; Macaques; i.m.	-	[57]
	NYVAC + ALVAC				Protein	[58]
	NYVAC (-C-KC)	gp140, ZM96	Tier 1	10^8^ pfu; Macaques; i.m.	Protein	[59]
	NYVAC	gp160 & gp120, HIV-2_SBL/ISY_, HIV-1_IIIB_	HIV-2_SBL6669_	10^7^ pfu; Macaques; s.c. i.m.	Protein	[60,61,62]
	Tiantan vaccinia	gp140 & gp145, cn54	HIV-1 primary isolates	10^7^ pfu; mice & guinea-pigs; i.m.	DNA	[63,64]
		gp140, cn54	Homologous (clade C); Heterologous (clade B)	5 × 10^5^–1 × 10^7^ pfu; Macaques; i.d.	DNA	[65]
	MVA	gp150, T/F ^ix^	Tier 2	10^8^ TCID_50_; Macaques; i.m.	DNA & protein	[66]
		gp150, SIV239	Tier 1			[67]
		gp140, clade B ADA	HIV_MN_	10^8^ pfu; Guinea-pigs; i.d. i.m.	-	[68]
	Fowlpox	gp160, 89.6P	Homologous	5 × 10^7^ pfu; rabbits; i.d.	DNA	[69]
	Vaccinia (+ SeV)	gp160, JR-CSF	Tier 1	Vaccinia: 10^7^ pfu s.s./SeV: 4 × 10^7^ ciu i.n.; mice	DNA	[70]
	Vaccinia	gp140, BH10 & chimeric (V1–V5)	HIV_MN_	10^8^ pfu; rabbits; i.d.	DNA	[71]
Adeno-virus	SAd4 ^x^	gp160, gp140, gp120; 1086.C	Tier 1 clade C; tier 2 clade C	10^11^ vp; rabbits; i.m. & i.n.	Protein	[72]
	Ad5hr ^xi^	rhFLSC (gp120: D1+D2); BaL ^xii^	Homologous; tier 2 clades B and D; SHIV_SF162P4_	5 × 10^8^ pfu; macaques; i.n. oral, i.t.	Protein	[73]
	Ad5	gp120, HIV-1_Bx08_	HIV-1_MN_	4.4 × 10^11^ pfu; macaques; i.m.	Protein	[74]
		gp140, HxB2/BaL & 89.6P	Homologous	10^12^ vp; macaques; i.m.	DNA	[75,76]
	Ad5/35	gp160, HIV_IIIB_	HIV-1_LAI_	10^10^–10^11^ vp; mice & macaques; i.m	DNA	[77]
	Ad4 + SAd7	gp150, 1086.C	Tier 1	0.5 × 10^11^ vp; i.n. and 10^11^ vp; i.m.; macaques	Protein	[78]
	Ad5 & 7	gp160, HIV_MN_	South African subtype C (TV-1)	10^7^–10^9^; chimpanzee; i.n.	Protein	[79]
	Ad4, 5, 7	HIV-1 gp160	HIV_IIIB_, HIV_SF2_, HIV_MN_	10^7^ pfu; chimpanzees; i.n.	Protein	[80]
				10^9^ pfu; beagles; i.t.		[81]
	+ MVA	gp140, BG505 SOSIP.664	Tier 1A; tier 2	5 × 10^10^ vp ChAdOx1.BG505s, 10^8^ PFU MVA; rabbits; i.m.	Protein	[82]
		gp140, SIV_SME543_	Tier 1	2 × 10^10^ vp Ad26, 10^8^ pfu MVA; macaques; i.m.	DNA	[83]
		Mosaic Env/Gag/Pol	Tier 1; tier 2	Ad35&26: 4 × 10^10^ vp, MVA: 10^8^ pfu; macaques; i.m.	-	[84,85]
Parvovirus	AAV	gp160, HIV_IIIB_	HIV-1_BaL_	10^9^–10^11^ vp; mice; i.n. i.m. s.c. i.p.	AAV-IL-2	[86]
	AAVrh32.33 + SAdV24	gp140, HIV W61D	Homologous	10^12^ gc AAVrh32.33: 2 × 10^11^ vp SAdV24 HIV; macaques; i.m.	-	[87]
Paramyxo-virus	Newcastle disease	gp140 & gp160, HIV-1 BaL	Tier 1A & 1B; tier 2	2 × 10^5^–1 × 10^6^ pfu; guinea pigs; i.n.	Protein	[88,89]
	Measles	gp160 & gp140, HIV-1 89.6	Homologous; primary isolates	5 × 10^6^ TCID_50_; Mice; i.p.	-	[90]
Flavivirus	YFV17D	gp120, T/F	Tier 1	10^4^ pfu; Mice; s.c.	Protein	[91]
Lentivirus	Integrase defective	gp140, EnvC.1086	Tier 1	3 × 10^8^ tu; Macaques; i.m.	-	[92]
Matonavirus	Rubella RA27/3	TM1ΔV1–V3 core gp120, 426c	IIIB (clade B); CAP85 (clade C)	8 × 10^4^–1 × 10^5^ pfu; Macaques; i.m.	Protein	[93]
Picornavirus	Polio (Sabin type 1) ^xiii^	gp41, HIV-1	African isolates; SF2, SF33, HTLV-III_B_, HTLV-III_RF_	10^8^ TCID_50_; rabbits; i.d. s.c. i.m.	-	[94]
Togavirus	Semliki Forest virus	gp140, YU2	MN, HXb2, SF162, 89.6, JR-CSF	5 × 10^7^ ciu; rabbits; i.d. s.c. i.m.	Protein	[95]

^i^ VSV—vesicular stomatitis virus; VSV-GP—VSV with the glycoprotein of the lymphocytic choriomeningitis virus; NYVAC—New York vaccinia virus; NYVAC-C-KC—replication-competent NYVAC variant; ALVAC—Avian vaccinia; MVA—Modified-vaccinia-Ankara; Ad—Adeno; SeV—Sendai virus; AAV—Adeno-associated virus; YFV—Yellow fever vaccine; ^ii^ SHIV—simian/human immunodeficiency virus; HTLV-III—human T-cell lymphotropic virus type III; ^iii^ pfu—plaque forming units; ffu—focus forming units; TCID_50_—tissue culture infectious dose 50; vp—virus particles; gc—genome copies; tu—transducing units; ciu—cell infectious units; ^iv^ i.n.—intranasal; i.m.—intramuscular; i.p.—intraperitoneal; s.c.—subcutaneous; i.d.—intradermal; i.t.—intratracheal; s.s.—skin scarification; ^v^ gp140 fused to transmembrane (TM) domain and cytoplasmatic tail of VSV-G; ^vi^ gp120 fused to TM domain and cytoplasmatic tail of VSV-G; ^vii^ gp140 + TM HIV + cytoplasmatic tail of VSV-G or rabis-G; ^viii^ removal of the immunomodulatory viral molecule B19; ^ix^ T/F—transmitted/founder; ^x^ SAd—replicating simian Ad vector; ^xi^ hr—host range mutant (allows growth in monkey cells); ^xii^ Full-length single-chain HIV-1_BaL_ gp120 linked to the D1 and D2 domains of rhesus macaque CD4; ^xiii^ Virus in complete Freund’s adjuvant.
